# Supplementary material for: Integration of Chemometrics and Sensory Metabolomics to Validate Quality Factors of Aged Baijiu (Nianfen Baijiu) with Emphasis on Long-Chain Fatty Acid Ethyl Esters
Source: Foods. 2023 Aug 17;12(16):3087. doi: 10.3390/foods12163087 (PMC10453570; doi:10.3390/foods12163087)
Supplement: Supplementary file 1 [file foods-12-03087-s001.zip › foods-2540326-supplementary.pdf]

Table S1. Distribution of LCFAEEs in sample groups.

Numbering of Baijiu Sample Groups	ET (mg/L)	EP (mg/L)	EO (mg/L)	9-EO (mg/L)	912-EO (mg/L)
B-1993	0.41 ± 0	4.28 ± 0.01	0.96 ± 0	1.26 ± 0.01	1.4 ± 0.07
B-2003	0.52 ± 0.01	7.82 ± 0.01	1.01 ± 0.01	2.44 ± 0.01	3.48 ± 0.09
B-2008	0.37 ± 0	3.48 ± 0.02	0.96 ± 0.01	1.3 ± 0.12	1.54 ± 0.03
B-2013	0.33 ± 0	1.97 ± 0.03	0 ± 0	1.15 ± 0.01	1.13 ± 0.03
B-2014	0.37 ± 0.01	3.47 ± 0	0.97 ± 0	1.39 ± 0.03	1.5 ± 0.07
B-2015	0.35 ± 0	2.84 ± 0.01	0 ± 0	1.23 ± 0.01	1.42 ± 0.01
B-2017	0.36 ± 0.01	2.68 ± 0.01	0 ± 0	1.15 ± 0.01	1.24 ± 0
B-2018	0.37 ± 0	3.57 ± 0.01	0 ± 0	1.28 ± 0.01	1.35 ± 0.01

Table S2. Examining the correlation between LCFAEEs and sensory evaluation scores.

Name	Evaluation Criterion	Fruity Aroma	Floral Aroma	Sweet-Smelling	Grain Fragrance	Aged Fragrant	Initial Gustatory Impression	Olfactory Sensation in the Oral Cavity	Mid-laryngeal Gustatory Perception	Off-Flavor	Irritation
ET	Pearson correlation	0.633	0.698	0.271	−0.050	0.680	0.245	0.293	0.740*	0.491	0.507
	Sig.	0.092	0.054	0.517	0.907	0.063	0.559	0.481	0.036	0.216	0.199
EP	Pearson correlation	0.645	0.707*	0.238	−0.006	0.671	0.182	0.242	0.694	0.522	0.471
	Sig.	0.084	0.050	0.571	0.989	0.068	0.666	0.563	0.056	0.184	0.239
EO	Pearson correlation	0.274	0.446	0.640	−0.109	0.750 *	0.329	0.417	0.731*	0.225	0.159
	Sig.	0.512	0.268	0.087	0.798	0.032	0.426	0.304	0.040	0.593	0.707
EO9	Pearson correlation	0.467	0.628	0.242	−0.186	0.446	−0.047	0.050	0.703	0.617	0.384
	Sig.	0.243	0.095	0.564	0.659	0.268	0.912	0.906	0.052	0.103	0.347
EO912	Pearson correlation	0.486	0.641	0.267	−0.129	0.442	−0.040	0.076	0.721*	0.545	0.414
	Sig.	0.222	0.086	0.522	0.761	0.273	0.925	0.859	0.044	0.162	0.308

* It indicates that at the level of 0.05, the correlation is significant.
